# Supplementary material for: Inhibition of Metastatic Hepatocarcinoma by Combined Chemotherapy with Silencing VEGF/VEGFR2 Genes through a GalNAc-Modified Integrated Therapeutic System
Source: Molecules. 2022 Mar 24;27(7):2082. doi: 10.3390/molecules27072082 (PMC9000533; doi:10.3390/molecules27072082)
Supplement: Supplementary file 1 [file molecules-27-02082-s001.zip › molecules-1634250-supplementary.pdf]

Supporting Information

Inhibition of Metastatic Hepatocarcinoma by Combined Chemotherapy with Silencing VEGF/VEGFR2 Genes through GalNAc-Modified Integrated Therapeutic System

Xunan Li, Nian Liu, Qiuyu Wang, Xiang Wang and Jing Hu*

Experimental section

Synthesis of 3-azido-2-hydroxypropyl methacrylate (AHPMA): TBA-N_3 (16.2 g, 57.0 mmol), $NaHCO_3$ (3.8 g, 45.2 mmol) and 60 ml THF/H_2O mixed solvent (5:1, v v^{-1}) were charged in a 150 mL round-bottomed flask, and then glycidyl methacrylate (GMA) (5.4 g, 37.8 mmol) was added slowly into the mixture by syringe with stirring. [1,2] The mixture was stirred at room temperature for 48 h. After removing the insoluble salts by filtration, the reaction solution was evaporated by rotary evaporation. The residue was extracted twice by DCM and the combined organic phase was dried over Na_2SO_4. The solvent was removed and the product was further purified by silica column chromatography using hexane/ethyl acetate (9:1, v v^{-1}) as the eluent, to afford 5.01 g of the targeted substance AHPMA (yield: 71%). ^{1}H NMR (400MHz, CDCl3, δ): 6.11 (s, J = 7.7 Hz, 1H; CH2=C-), 5.59 (s, J = 7.8 Hz, 1H; CH2=C-), 4.19 (dd, 2H; -COOCH_2-), 4.04 (m, 1H; -CHOH), 3.38 (m, 2H; -CH_2N_3), 2.89 (br, 1H; -OH), 1.91 (m, 3H; -CH_3). ESI-MS m/z: $[M+Na]^+$ calcd for $C_7H_{11}N_3O_3$, 208.18; found, 208.07. The results are identical to the published results.

Synthesis of RhB-based initiator (RhB-Br): RhB-Br was prepared according to a previous protocol.[4] Ethylene glycol (15.5 g, 250.0 mmol) and triethylamine (1.4 mL, 10.0 mmol) were added into a round-bottom flask equipped with a magnetic stirring bar. [3] After cooling to 0 °C in an ice-water bath, 2-bromoisobutyryl bromide (1.2 mL, 9.7 mmol) was added dropwise over 30 min. After stirring at room temperature for 3 h, mixture was quenched with 1 L of H_2O and extracted with DCM (100 mL×3). The combined organic phase was further extracted with ddH_2O (100 mL×3). After drying over anhydrous Na_2SO_4, DCM was removed on a rotary evaporator. The residues were purified by distillation (85°C, 30mTorr) to yield a viscous, clear, and colorless liquid 2-hydroxyethyl 2-bromoisobutyrate (1.5 g, 72%). Then, RhB-Br was synthesized by adding RhB (4.8 g, 10.0 mmol), EDC·HCl (2.9 g, 15.1 mmol), 2-hydroxyethyl 2-bromoisobutyrate (3.2 g, 14.9 mmol), and anhydrous DCM (40 ml) into a round-bottom flask equipped with a magnetic stirring bar. After cooling to 0 °C in an ice-water bath, 4-dimethylaminopyridine (1.8 g, 15.0 μmol) was added. The reaction was stirred at room temperature for 12 h. The reaction mixture was then sequentially extracted with 0.1 M HCl (50 mL×3), aqueous saturated $NaHCO_3$ (50 mL×3), and aqueous saturated NaCl solution (50 mL×3). After drying over anhydrous Na_2SO_4, DCM was removed on a rotary evaporator. The crude product was purified by column chromatography (DCM/methanol = 10:1, v v^{-1}), yielding a purple powder (4.0g, 62 %). 1H NMR (400MHz, 4 $CDCl_3$, δ): 8.27 (t, J = 8.4 Hz, 1H; aromatic protons), 7.81 (t, J = 7.5 Hz, 1H; aromatic protons), 7.72 (t, J = 7.7 Hz, 1H; aromatic protons), 7.29 (d, J = 7.6 Hz, 1H, aromatic protons), 7.03 (m, 2H; aromatic protons), 6.90 (m, 2H; aromatic protons), 6.78 (m, 2H), 4.28 (m, 4H;- CH2-CH2-), 3.62 (q, J = 7.3 Hz, 8H; CH3-CH2-), 1.84-1.68 (m, 6H; -CH3), 1.30 (m, 12H; CH3CH2-). ESI-MS m/z: $[M + Na]^+$ calcd for $C_{34}H_{40}BrN_2O_5^+$, 658.21; found, 658.23. The results are identical to the published results.

Preparation of N-acetyl galactosamine: To a stirred solution of tetra-O-acetate -GalNAc (0.60 g, 1.54 mmol) in dry DCM (50 mL) was added propargyl alcoahol (182 μL, 3.08 mmol) and Yb (OTf)3 (0.286 g, 0.46 mmol) before the temperature was raised to cause reflux. [4] The reaction was maintained at this temperature for 24 hours before it was washed with H_2O (20 mL), the organic layer dried ($MgSO_4$), and concentrated. Column chromatographic purification (silica, AcOEt) gave the desired - glycoside (516 mg, 87%) as a colourless solid. NaOMe (63 μL, 0.06 mmol) was added to a solution of the above solution (222 mg, 0.58 mmol) in MeOH (10 mL) at room temperature. After 4 h, the reaction mixture was neutralized with Dowex50W-X8 (H^+) resin.

Preparation of micelles: Solvent exchange method was applied to prepare the micelles.[5] The polymer (10.0 mg) was first dissolved in DMSO (0.5 mL) under vigorous stirring. The polymer solution was diluted by dropwise addition of 5.0 mL of ddH_2O. The solution was placed into a dialysis bag (MWCO 3500) and dialyzed against ddH_2O for 2 days with exchange of ddH_2O every 6 h. Finally, the micelle solution was filtered with a 0.45 μm microporous filter to eliminate the aggregated micelles. The freshly prepared micelles were further characterized by TEM and DLS.

Preparation of 5-FU/siRNA@GalNAc-pDMA: GalNAc-pDMA was synthesized and modified via metalla-click reaction according to the modified protocol (see supporting information). Then 5-FU and triethylamine were dissolved in DMSO, and stirred at room temperature for 2 h. The solution was then dialyzed for 48 h to obtain 5-FU/@GalNAc-pDMA particles. VEGF-siRNA and VEGFR2-siRNA was then condensed. Equal volumes of 5-FU@GalNAc-pDMA and VEGF-siRNA/VEGFR2-siRNA solutions in RNase free buffer were mixed to form 5-FU/siRNA@GalNAc-pDMA complexes, where the solutions were mixed and kept for 30 min at room temperature.

Drug loading measurement: The triethylamine was added slowly to a mixture of polymers p(RhB-DMAEMA-AHPMA-GalNAc) and 5-FU in DMSO and stirred for 5 h. Then 5ml double distilled water was added drop by drop and stirred for another 30 min, followed by stirring in double distilled water for 48 h at room temperature and the water was replaced every 6 h. The loading content (LC) and Encapsulation Efficiency (EE) of 5-FU were determined by absorption spectrophotometry at 265 nm. The LE and EE of 5-FU were analyzed according to following formula:
LE (%) = (weight of loaded 5-FU) / (total weight of copolymer) ×100%
EE (%) = (weight of loaded 5-FU) / (weight of fed drug initially) ×100%

Hemolysis assay: To perform hemolysis assay, 500 μL of GalNAc-pDMA solutions (60-600 μg mL^{-1}) were mixed with 500 μL of fresh goat red blood cells and incubated at 37°C for 2 h. The mixture was centrifuged (1000 g, 20 min) and the supernatants (100μL per well) were then transferred to a 96- well plate for an optical measurement at the wavelength of 540 nm. The positive control was sterilization deionized water, and the negative control was DMEM medium without phenol red.
Hemolysis (%) = $(OD_{Sample} - OD_{Negative\ control}) / (OD_{Positive\ control} - OD_{Negative\ control}) \times 100\%$

Establishment of HCC-bearing mice models

Animal experiments were approved by Technology Animal Care and Use Committee of Jiangnan University (Appr. Nr.). The healthy male nude mice (BALB/c, 18-20 g, 4 weeks old) were from Slac Laboratory Animal Co. Ltd (Hunan, China). To obtain subcutaneous HCC-bearing mice, 6.0×10^6

of C5WN1 cells (100 µL in PBS/Matrigel (v:v, 1:1)) were inoculated subcutaneously (s.c.) into the hind leg flanks and tumor sizes were monitored every 2 days. The tumors were allowed to grow until the size reaches 50 mm^3. The tumor volume of each mice was calculated by to the formula: Tumor volume = 0.5236 × (tumor length) × $(\text{tumor width})^2$.

References

[1] Lin, Shaoliang, Cai, Xin, Zhong, Liang, Yue, Xiaohua, Polymer chemistry, 6 (2015) 3875,https://doi.org/10.1039/C5PY00234F.

[2] C. Gao, X. Zheng, Soft Matter, 5 (2009) 4788-4796,https://doi.org/10.1039/B909994H.

[3] J. Cai, Y. Yue, D. Rui, Y. Zhang, S. Liu, C. Wu, Marcromolecules 2011, 44, 2050.

[4] R.L. Siegel, K.D. Miller, H.E. Fuchs, A. Jemal, CA-A CANCER JOURNAL FOR CLINICIANS, 71 (2021) 7-33,https://doi.org/10.1039/C5OB01870F.

[5] M. Karayianni, S. Pispas, in Fluorescence Studies of Polymer Containing Systems, Vol. 16 (Eds: K. Procházka), Springer International Publishing, Cham, Switzerland 2016.

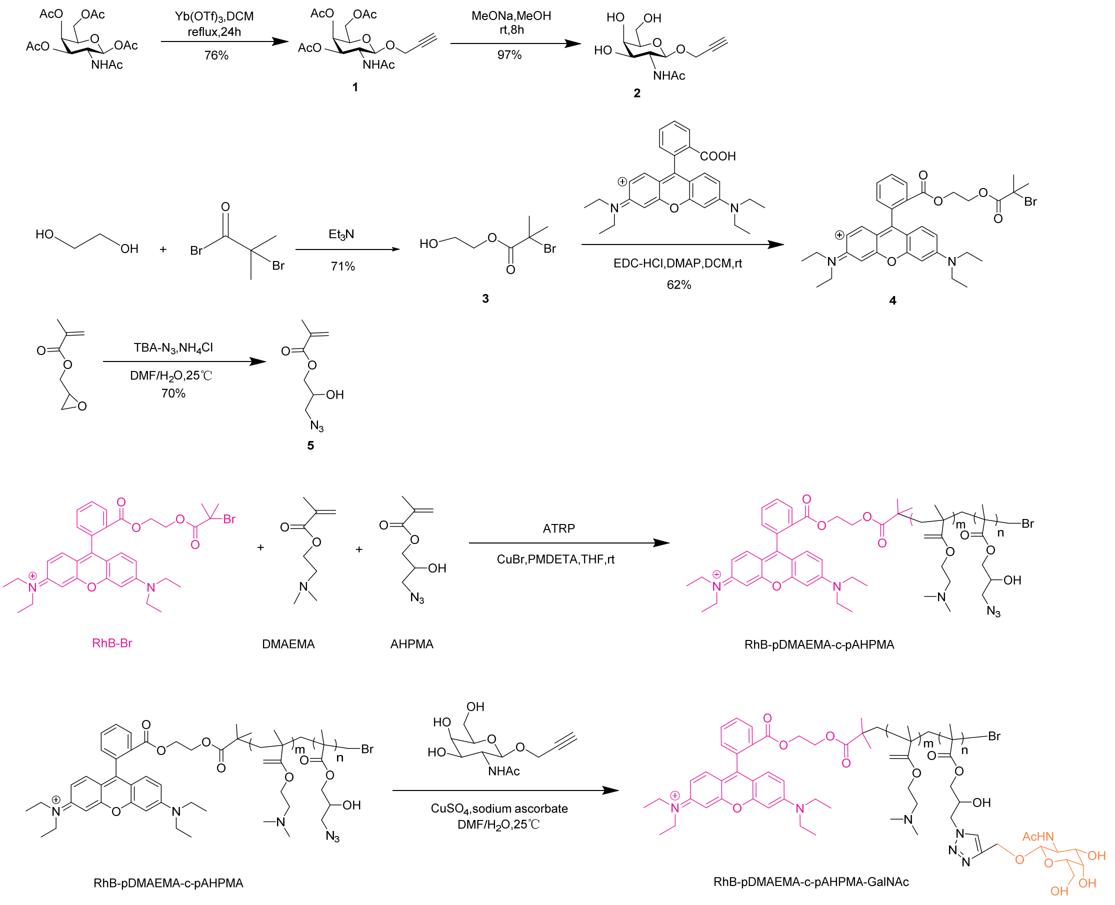

Scheme S1. Schematic illustration of the synthesis route of RhB-pDMAEMA-pAHPMA-GalNAc.

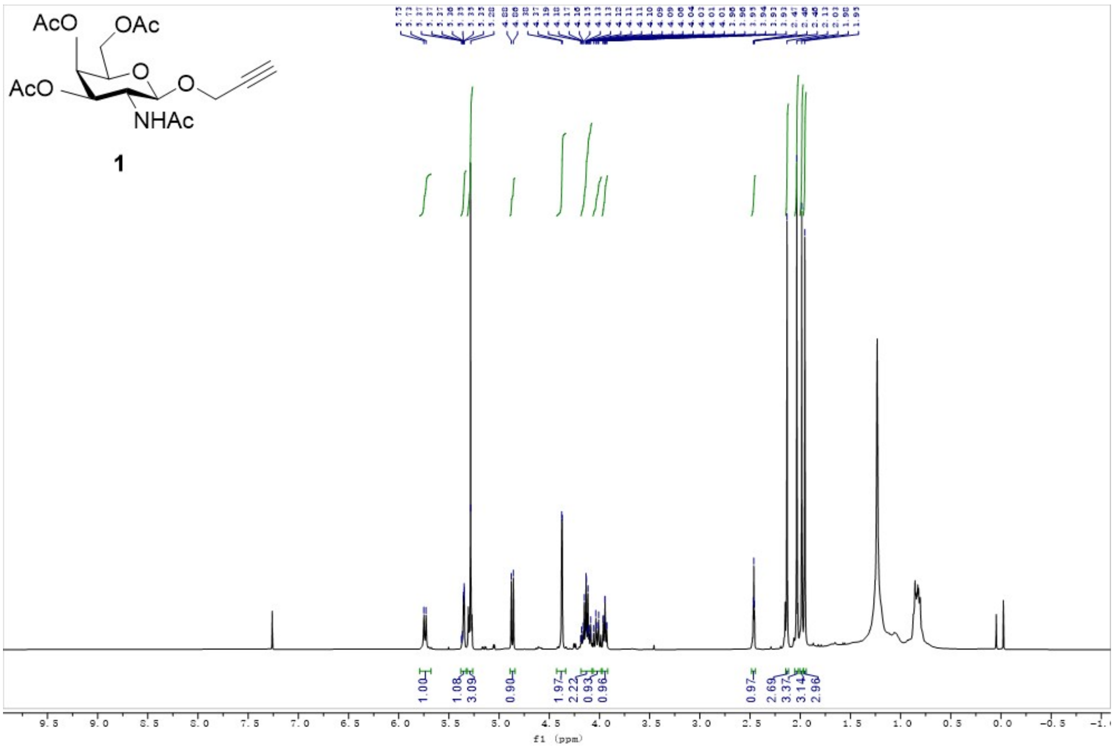

Figure S1. ^{1}H NMR ($CDCl_3$, 400MHz) of compound 1.

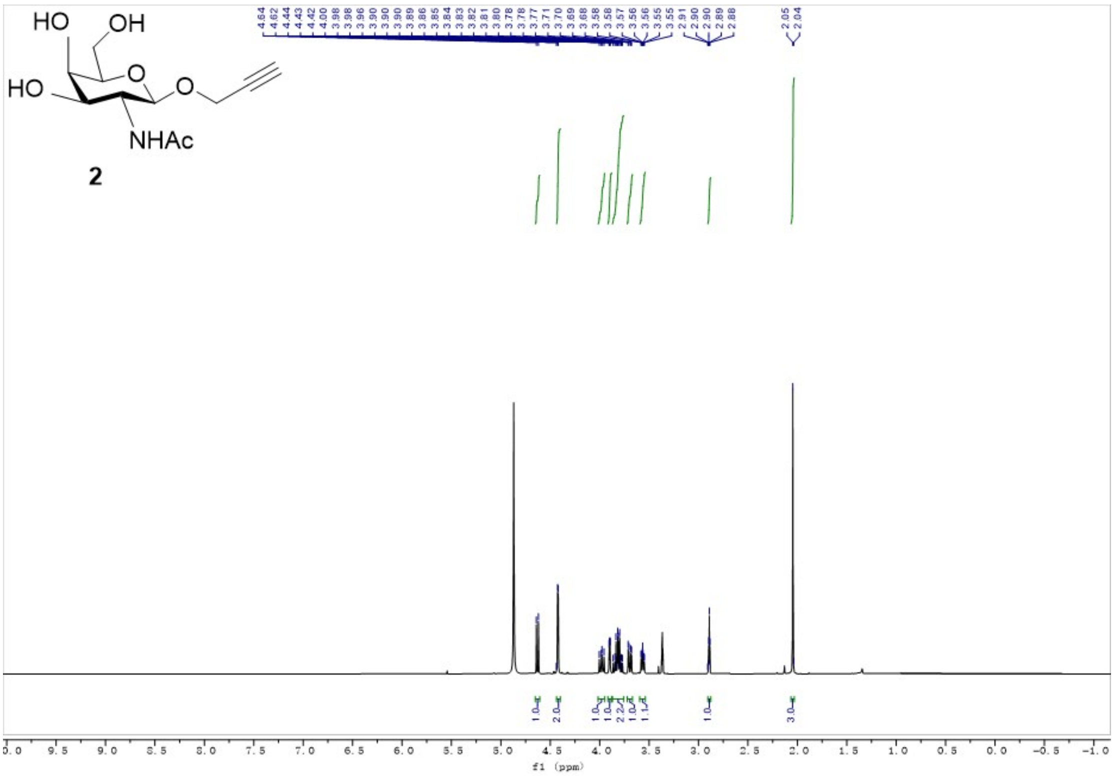

Figure S2. ^{1}H NMR ($CDCl_3$, 400MHz) of compound 2.

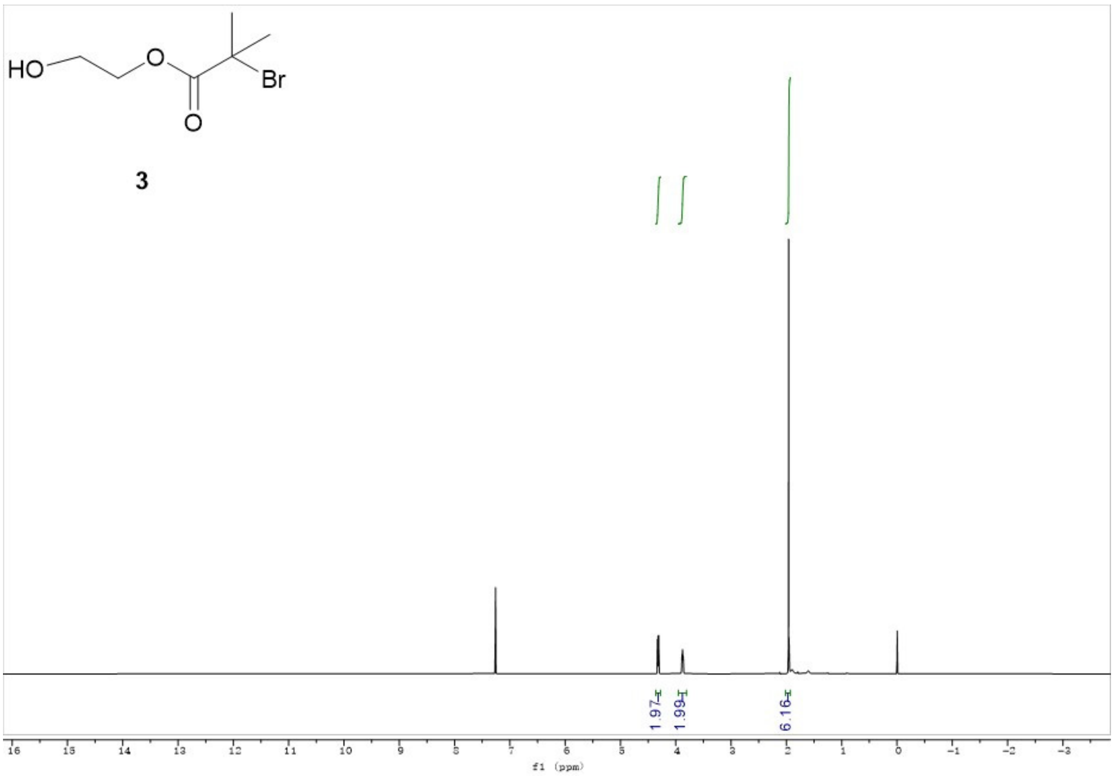

Figure S3. **^{1}H NMR ($CDCl_3$, 400MHz) of compound 3.**

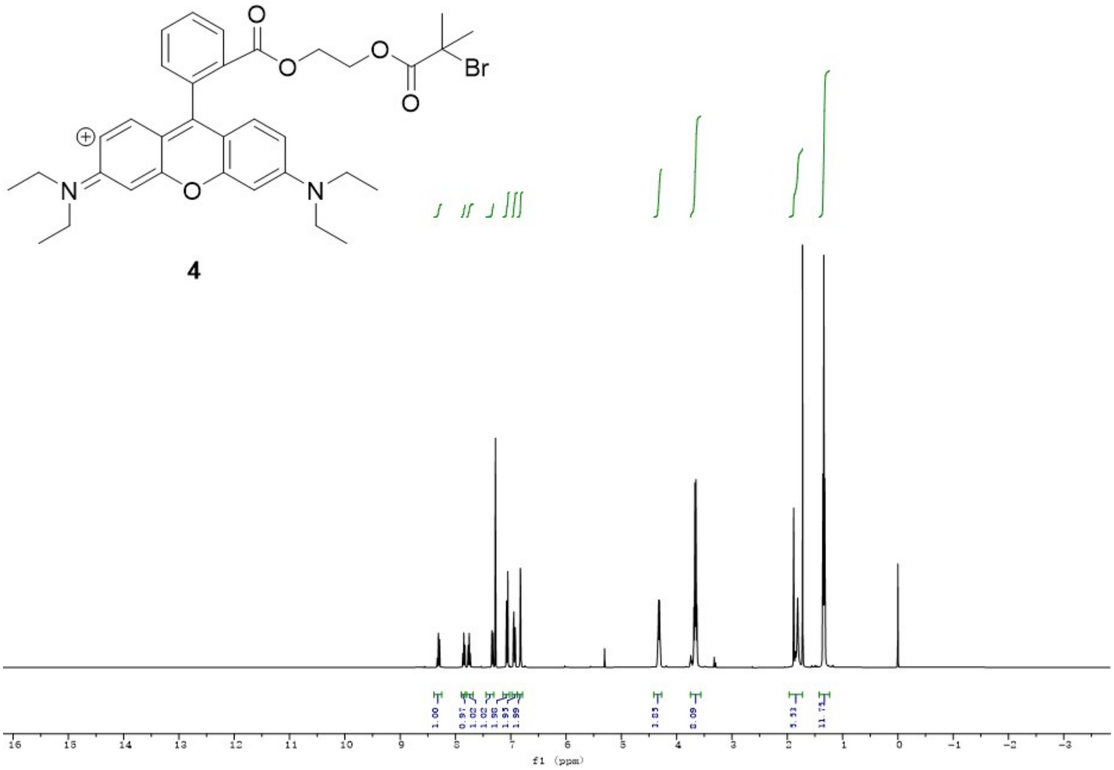

Figure S4. **^{1}H NMR ($CDCl_3$, 400MHz) of compound 4.**

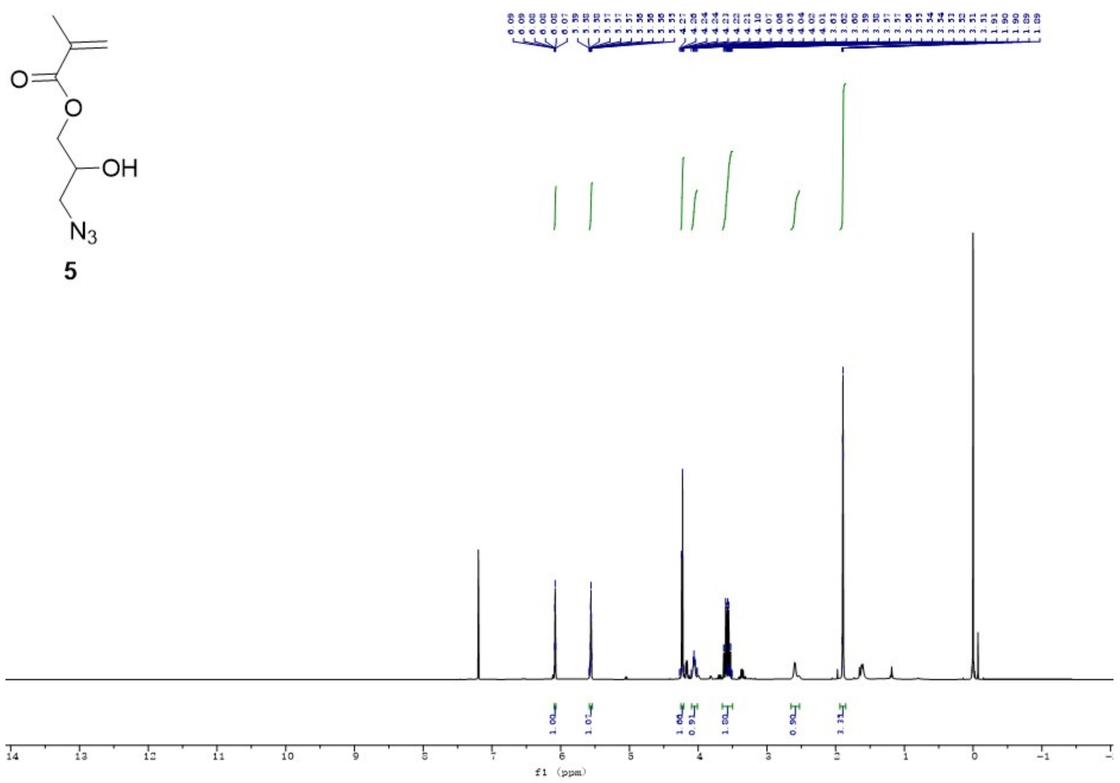

Figure S5. ^{1}H NMR ($CDCl_3$, 400MHz) of compound 5.

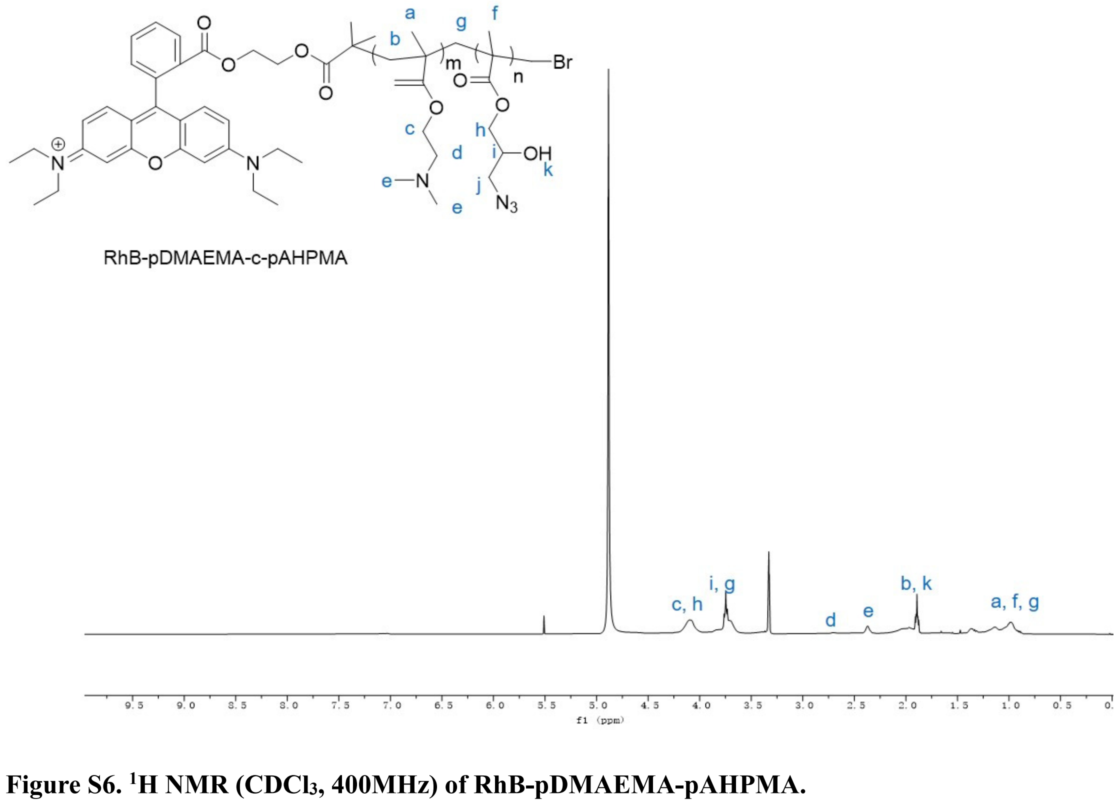

Figure S6. ^{1}H NMR ($CDCl_3$, 400MHz) of RhB-pDMAEMA-pAHPMA.

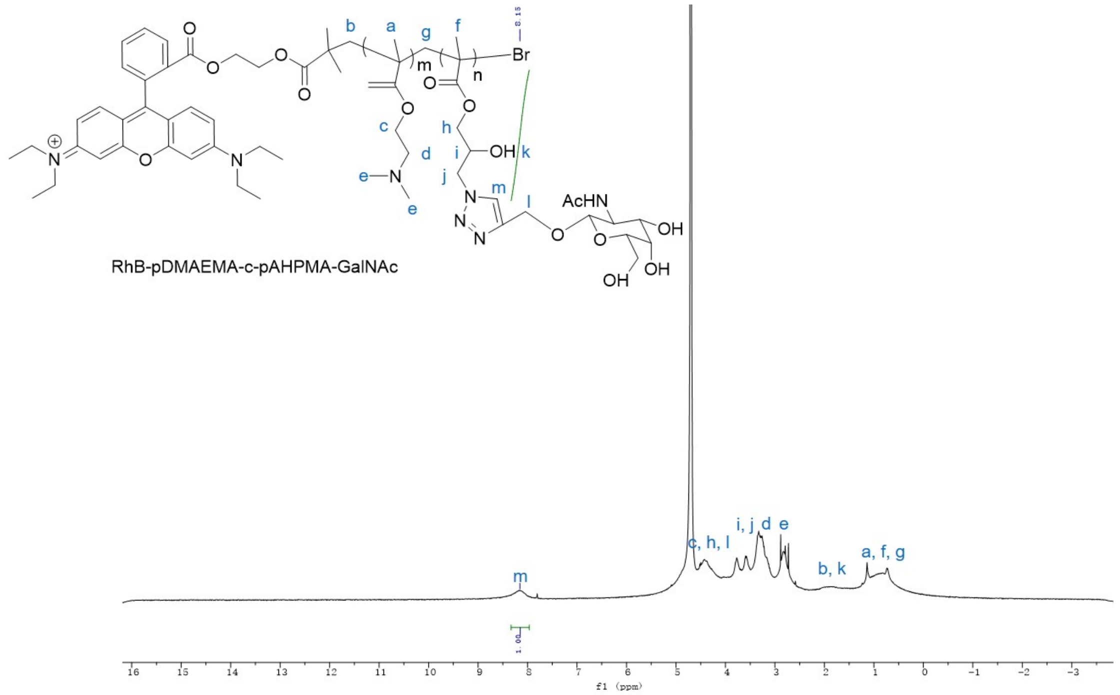

Figure S7. ^{1}H NMR ($CDCl_3$, 400MHz) of RhB-pDMAEMA-pAHPMA-GalNAc.

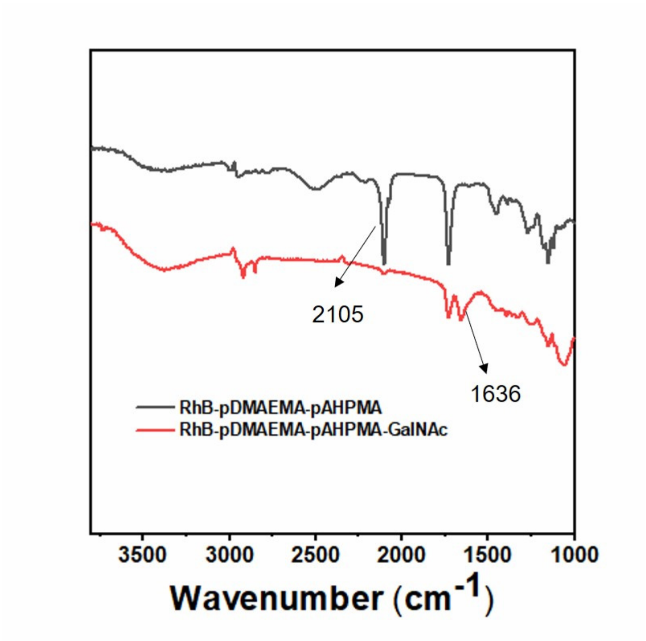

Figure S8. FT-IR spectrum of RhB-pDMAEMA-pAHPMA and RhB-pDMAEMA-pAHPMA-GalNAc.

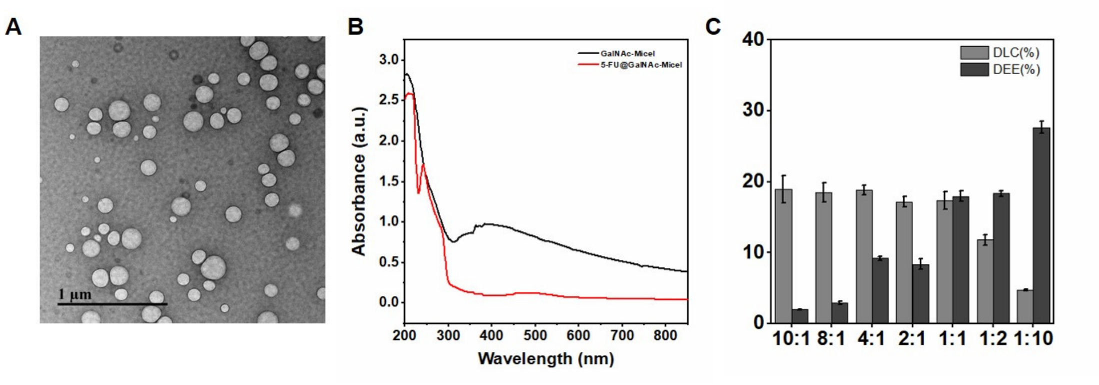

Figure S9. (A)TEM image of GalNAc-pDMA. (B) UV-vis absorption spectra of GalNAc-pDMA and 5-FU@GalNAc-pDMA. (C) Drug loading efficiency and encapsulation efficiency of 5-FU@GalNAc-pDMA.

Table S1. Sequences of VEGF-siRNA and VEGFR2-siRNA.

Strand	Sense sequence (5'-3')	Anti-sense sequence (5'-3')
VEGF-siRNA	*GG*AGUACCCUGAUGAGAUC*UU*	*GA*UCUCAUCAGGGUACUCC*UU*
VEGFR2-siRNA	GGUAAAGAUUGAUGAAGAAdTdT	UUCUUCAUCAAUCUUUACCdTdT

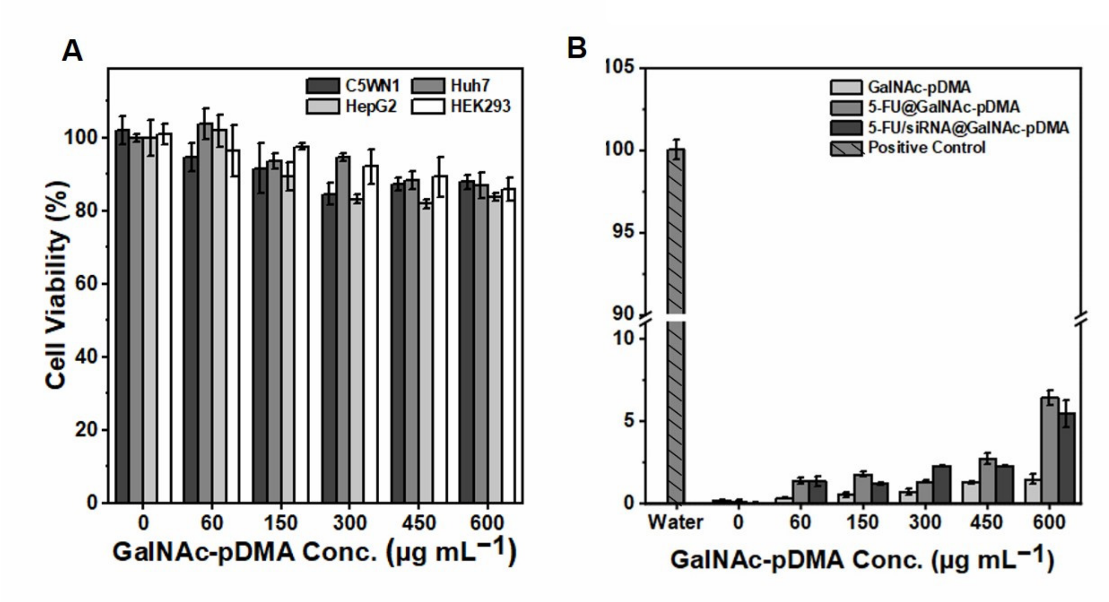

Figure S10. (A) Cell viability of HepG2, Huh7, C5WN1 and HEK293 cells incubated with GalNAc-pDMA at various concentrations for 48h. (B) Hemolysis test results of GalNAc-pDMA, 5-FU@GalNAc-pDMA and 5-FU/siRNA@GalNAc-pDMA. The error bars were obtained from six independent measurements.

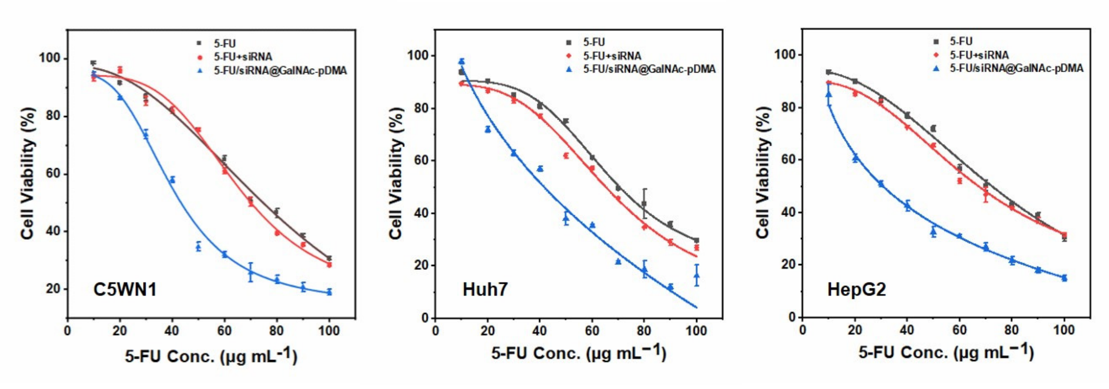

Figure S11. Inhibitory effect of different concentrations of 5-FU, 5-FU@GalNAc-pDMA, and 5-FU/siRNA@GalNAc-pDMA (5-FU at 10-100 μg mL^{-1}, GalNAc-pDMA at 60-600 μg mL^{-1}, and siRNA at 0.7-7 μg mL^{-1}) were tested with C5WN1, Huh7 and HepG2 cells, respectively, in DMEM medium for 48 h.

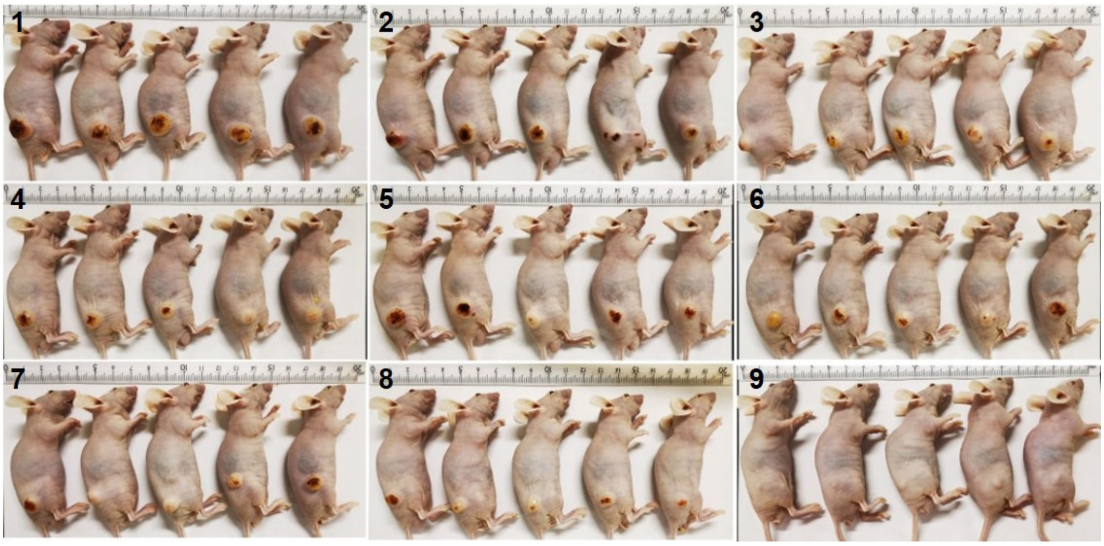

Figure S12. Morphological observation of subcutaneous HCC-bearing mice at the end of the treatment experiment. Mice were treated with 1) saline, 2) GalNAc-pDMA, 3) siRNA, 4) 5-FU, 5) 5-FU+siRNA, 6) siRNA@GalNAc-pDMA, 7) 5-Fu@GalNAc-pDMA, 8) 5-FU/siRNA@Glc-pDMA and 9) 5-Fu/siRNA@GalNAc-pDMA.

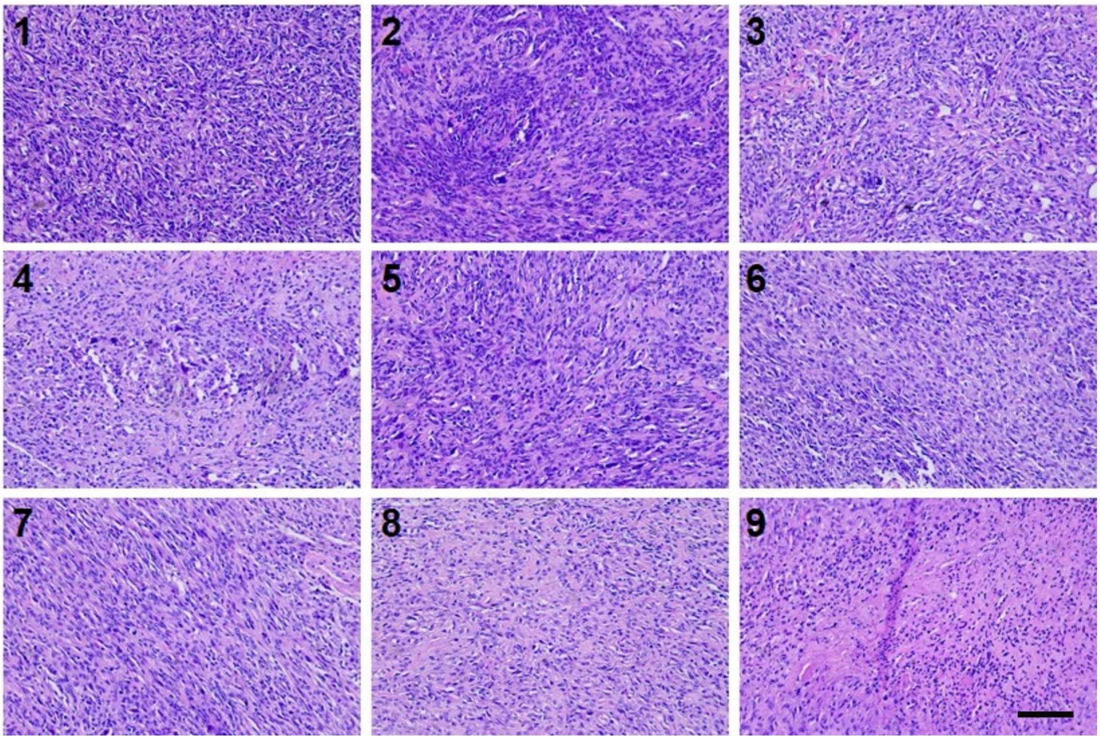

Figure S13. H&E staining of tumor sections after various treatments with 1) saline, 2) GalNAc-pDMA, 3) siRNA, 4) 5-FU, 5) 5-FU+siRNA, 6) siRNA@GalNAc-pDMA, 7) 5-Fu@GalNAc-pDMA, 8) 5-FU/siRNA@Glc-pDMA and 9) 5-Fu/siRNA@GalNAc-pDMA. Scale bars: 200 µm.

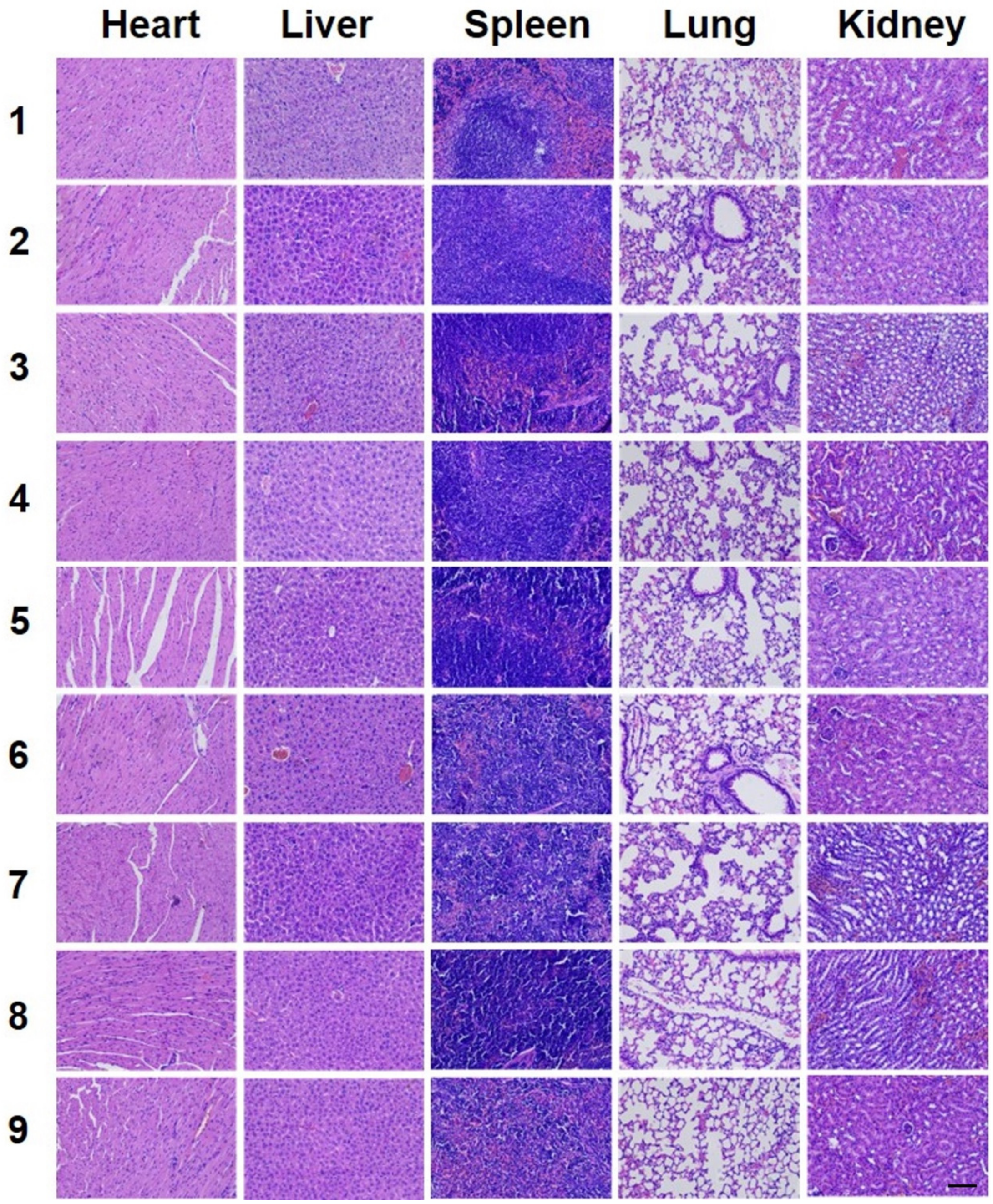

Figure S14. H&E staining of major organs in the subcutaneous HCC-bearing mice after the treatment experiment.1) saline, 2) GalNAc-pDMA, 3) siRNA, 4) 5-FU, 5) 5-FU+siRNA, 6) siRNA@GalNAc-pDMA, 7) 5-Fu@GalNAc-pDMA, 8) 5-FU/siRNA@Glc-pDMA and 9) 5-Fu/siRNA@GalNAc-pDMA. Scale bar: 200 µm.
